# Reciprocated tachycardias in cardiac laminopathy: a clinical case report

**DOI:** 10.1093/ehjcr/ytaf417

**Published:** 2025-08-28

**Authors:** Evgeny Zhelyakov, Natalia Sonicheva-Paterson, Svetlana Aleksandrova, Viktor Tcivkovskii, Andrei Ardashev

**Affiliations:** Department of Cardiovascular Surgery, Pirogov Russian National Research Medical University, bldg 6 Ostrovityaniva ulitsa, Moscow 117513, Russia; Tenaya Therapeuticals, Oyster Point Blvd., Suite 500. South San Francisco, CA 94080, USA; Magnetic Resonance Imaging Department, A. N. Bakulev National Medical Research Center for Cardiovascular Surgery, bld 135, Rublyovskoe shosse, Moscow 121552, Russia; Surgery Department, JSC Medicine, Bld 10 2-Tverskoy-Yamskoy pereulok, Moscow 125047, Russia; Feinberg School of Medicine, Northwestern University, 303E Chicago Ave, Ward 1-003, Chicago, IL 60611, USA

**Keywords:** AV reciprocated tachycardia, AV-node dual physiology, Laminopathy, Genetic cardiomyopathy, Myocarditis, Early indicator of laminopathy, Case report

## Abstract

**Background:**

Cardiac laminopathies, associated with mutations in the LMNA gene, are a rare inherited disorder characterized by a broad range of clinical manifestations. There are currently no data on the association between supraventricular re-entrant tachycardias and LMNA-related cardiomyopathy.

**Case summary:**

A 26-year-old male presented with either wide-QRS tachycardia with a left bundle branch block (LBBB) pattern or narrow QRS tachycardia, as well as a history of palpitations since age 15. Echocardiography showed no overt structural heart disease. Electrophysiological studies confirmed the diagnosis of orthodromic atrioventricular re-entrant tachycardia mediated by a concealed left posterolateral accessory pathway (AP), with transient LBBB and dual AV-node physiology, characterized by single echo beats. A short-run, asymptomatic episode of atrial fibrillation was induced. Cardiac magnetic resonance (CMR) imaging demonstrated myocardial hyperaemia, subepicardial late gadolinium enhancement, and a small pericardial effusion, initially interpreted as myocarditis according to the modified Lake Louise criteria. A family history of pacemaker implantation and cardiac death at age 65 of the patient’s grandfather, a history of re-entrant arrhythmia recurrence following ablation, and further CMR deteriorations led to genetic counselling. Genetic testing identified a heterozygous pathogenic variant in the LMNA gene (NM_170707.3.456_457insTCTC, NP_733821.1.Glu154GlnfsX2), classified as likely pathogenic and associated with laminopathy.

**Discussion:**

This case raises the question of whether the combination of re-entrant arrhythmias is coincidental or an early indicator of LMNA-related cardiomyopathy.

Learning points

- Supraventricular reciprocated tachycardias patients who exhibit multiple arrhythmias, a history of recurrence following ablation, suspicious family history, and imaging with subtle structural abnormalities may be early indicators of the genetic cardiomyopathy.
- Structural and electrical remodelling associated with laminopathies potentially may contribute to the re-entrant substrate for supraventricular re-entrant arrhythmias.
- Further research is required to explore the potential link between re-entrant arrhythmias and early-stage LMNA-related cardiomyopathy, guiding more targeted surveillance and therapeutic strategies.

## Introduction

Cardiac laminopathies, associated with mutations in the LMNA gene, are a rare (1:2500–5000) inherited disorder characterized by a broad range of clinical manifestations [heart failure (HF), sudden cardiac death (SCD), conduction block, ventricular tachycardias (VTs), and atrial fibrillation (AF)] in early- to mid-adulthood.^1–3^ There are currently no data on the association between atrioventricular re-entrant tachycardia (AVRT), atrioventricular nodal reciprocating tachycardia (AVNRT), and LMNA-related cardiomyopathy. We present the case of coexistence of both reciprocating tachycardias (RT) with intermittent left bundle branch block (LBBB). Issues related to the identification of relatively uncommon inherited cardiac disease in patients with the relatively common occurrence of supraventricular tachycardias (SVTs) are considered.

## Summary figure

**Figure:**
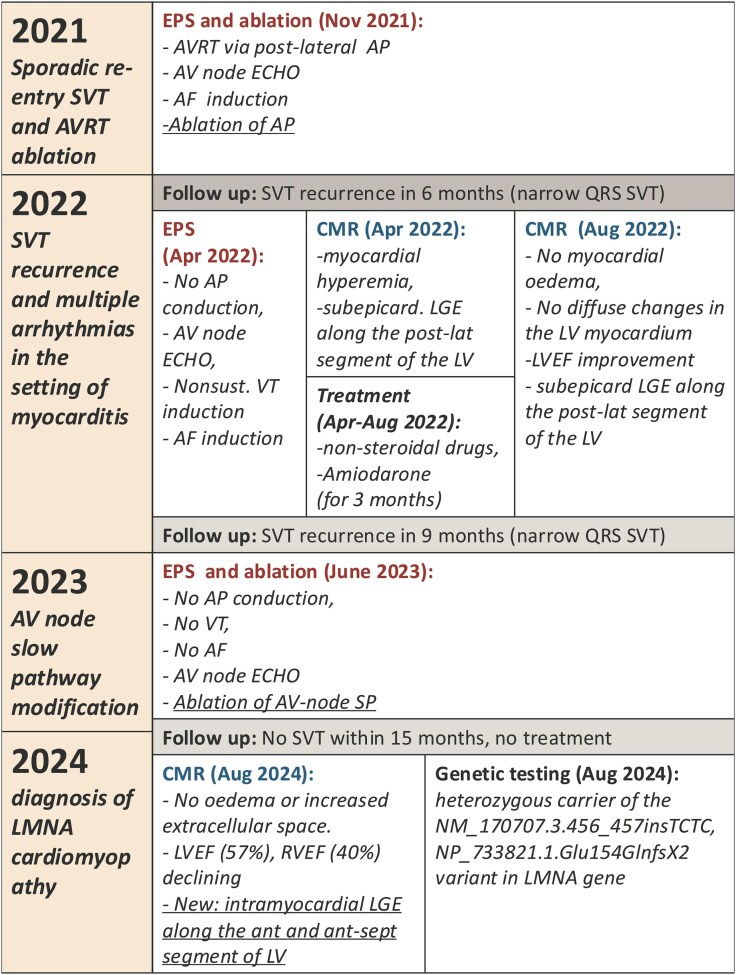
EPS, electrophysiological study; CMR, cardiovascular magnetic resonance; AV, atrioventricular; AP, accessory pathway; SP, slow pathway; SVT, supraventricular tachycardia; VT, ventricular tachycardia; AF, atrial fibrillation; LGE, late gadolinium enhancement; AVRT, atrioventricular re-entry tachycardia.

## Case presentation

A 26-year-old male was admitted due to recurrent episodes of narrow and wide-QRS tachycardia exhibiting a LBBB pattern without haemodynamic compromise (Summary figure). Family history was initially considered unremarkable, while 12-lead ECG in sinus rhythm (see *Supplement S1*) and echocardiography showed no overt structural heart disease.

### First stage—reciprocating tachycardias as a sporadic disease

**Figure 1:**
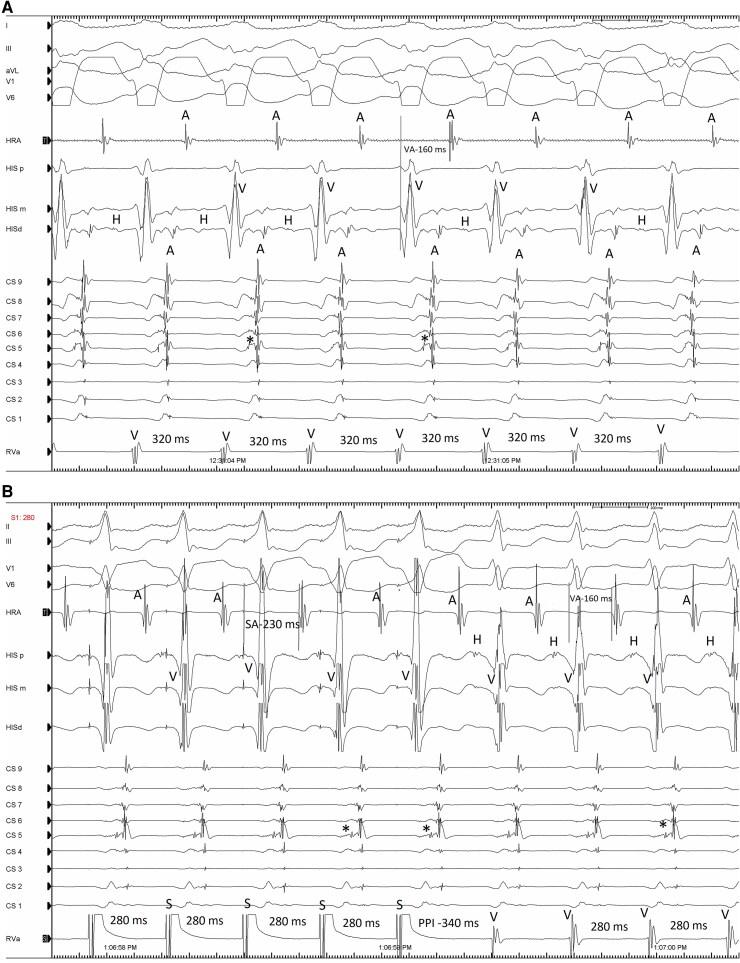

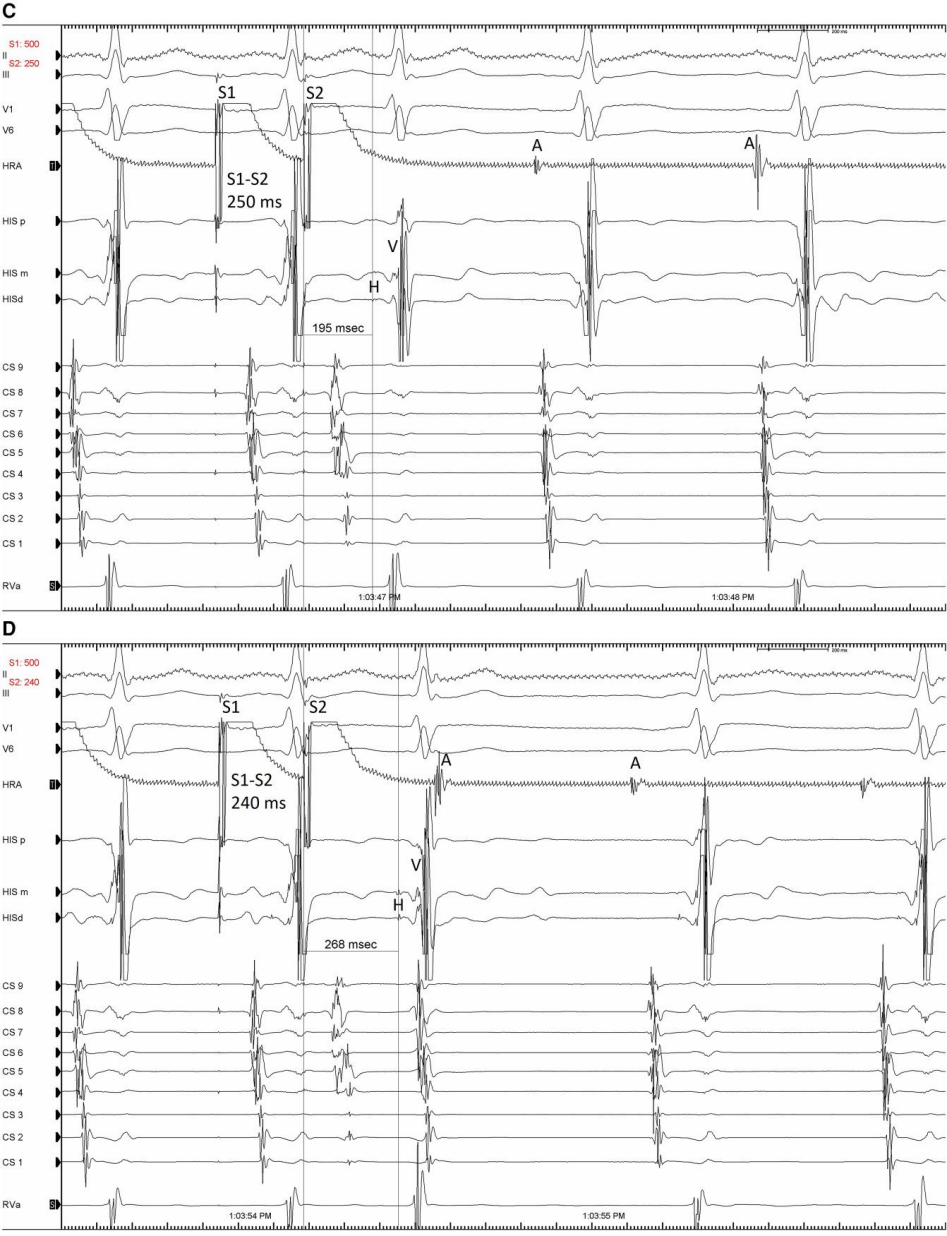
Clinical tachycardia and dual AV-node physiology. (*A*) Orthodromic AVRT with retrograde conduction via a left posterolateral accessory pathway (AP) (*) and a left bundle branch block (LBBB) pattern. (*B*) Entrainment from right ventricular apical pacing restores left bundle refractoriness and shortens the tachycardia cycle length (TCL) with V–A–V response and post-pacing interval (PPI) of 340 ms. The SA–VA difference < 85 ms and the PPI–TCL difference < 115 ms prove diagnosis of AVRT. (*C* and *D*) Dual AV-node physiology without atrioventricular nodal re-entrant tachycardia (AVNRT) during atrial programmed stimulation SA, stimulus to A interval; VA, VA interval during tachycardia; PPI, post-pacing interval; RVA, right ventricular apex; CS9 to CS1, proximal to distal coronary sinus; His p, His m, His d, His proximal, middle, and distal; HRA, high right atrium.

During the electrophysiological study (EPS), orthodromic AVRT with retrograde conduction via a left posterolateral AP (*Figure 1A* and *B*) and dual AV-node physiology were diagnosed. This was evidenced by a stereotypic jump in the AH-interval with single AV-node echoes, without AVNRT (*Figure 1C* and *D*). Additionally, a short, asymptomatic episode of AF lasting up to 2 min was observed. An AP was successfully eliminated using a transeptal approach without complications.

### Second stage—reciprocating tachycardias recurrence and multiple arrhythmias in the setting of myocarditis

Six months later, the patient experienced a recurrence of narrow QRS tachycardia with a cycle length of 280 ms, as confirmed by ECG. Atrioventricular re-entrant tachycardia and retrograde conduction through an AP were ruled out during the second EPS. Atrial S2 programming triggered nodal echoes without AVNRT. Ventricular S2 programming induced a non-sustained VT lasting four beats.

Due to the presence of multiple arrhythmias, we suspected underlying structural heart disease, such as myocarditis. However, laboratory tests (high-sensitivity cardiac troponin, N-terminal pro-B-type natriuretic peptide, and C-reactive protein) were normal. Cardiac magnetic resonance (CMR) showed myocardial hyperaemia, subepicardial late gadolinium enhancement (LGE) along the posterolateral segment of the left ventricle (LV), and a small pericardial effusion, which was interpreted as signs of myocarditis according to the modified Lake Louise criteria.^4^ The diagnosis of clinically suspected myocarditis was established,^5,6^ and the patient was treated with meloxicam 7.5 mg daily for three months.^5,6^

**Figure 2:**
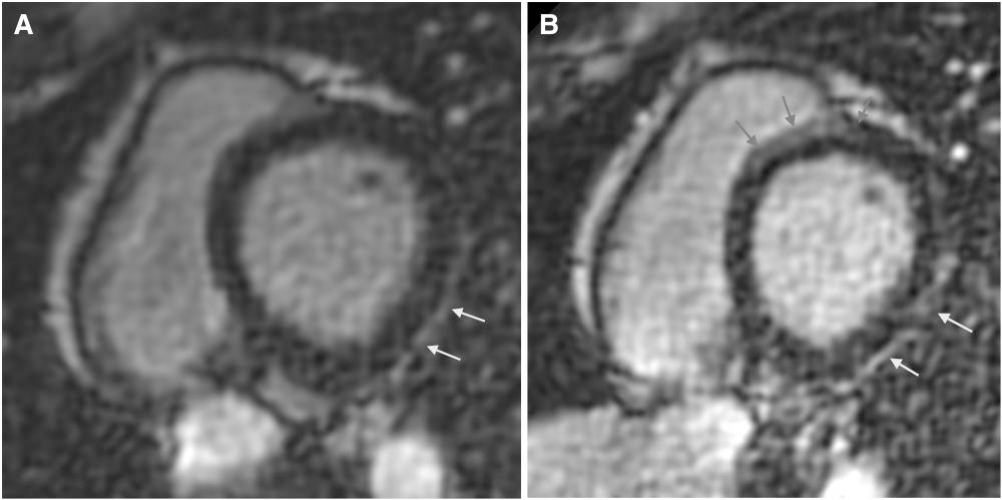
CMR during follow-up from 2022 to 2024. (*A*) 2022; (*B*) 2024. Delayed gadolinium enhancement (LGE) using phase-sensitive inversion recovery sequence, short-axis view. Subepicardial contrast enhancement of the posterolateral segment of the left ventricle (LV) is indicated by arrows. In 2024, new intramyocardial contrast enhancement appeared in the anteroseptal segment of the LV, marked by arrows.

Control CMR was performed one year later^4^ and showed an improvement in LV ejection fraction (LVEF) from 58% to 65%, with a decrease in LV end-diastolic volume (from 134 to 122 mL) and right ventricular (RV) end-diastolic volume (from 143 to 125 mL). The RV ejection fraction (RVEF) was at the lower limit of normal at 47%. The subepicardial LGE in the posterolateral segment of the LV remained unchanged (*Figure 2A*).

### Third stage—AV-node slow pathway modification

Because a few short SVT episodes were preserved in the 9-month post-op period, we conducted a third EPS. Programmed stimulation revealed only dual AV-node physiology without tachyarrhythmias. Following current guidelines,^7^ radiofrequency ablation (RFA) of the slow pathway was performed without complications. No tachyarrhythmia recurrences were observed during 15 months of follow-up.

### Fourth stage—firm diagnosis of genetic cardiomyopathy

**Figure 3:**
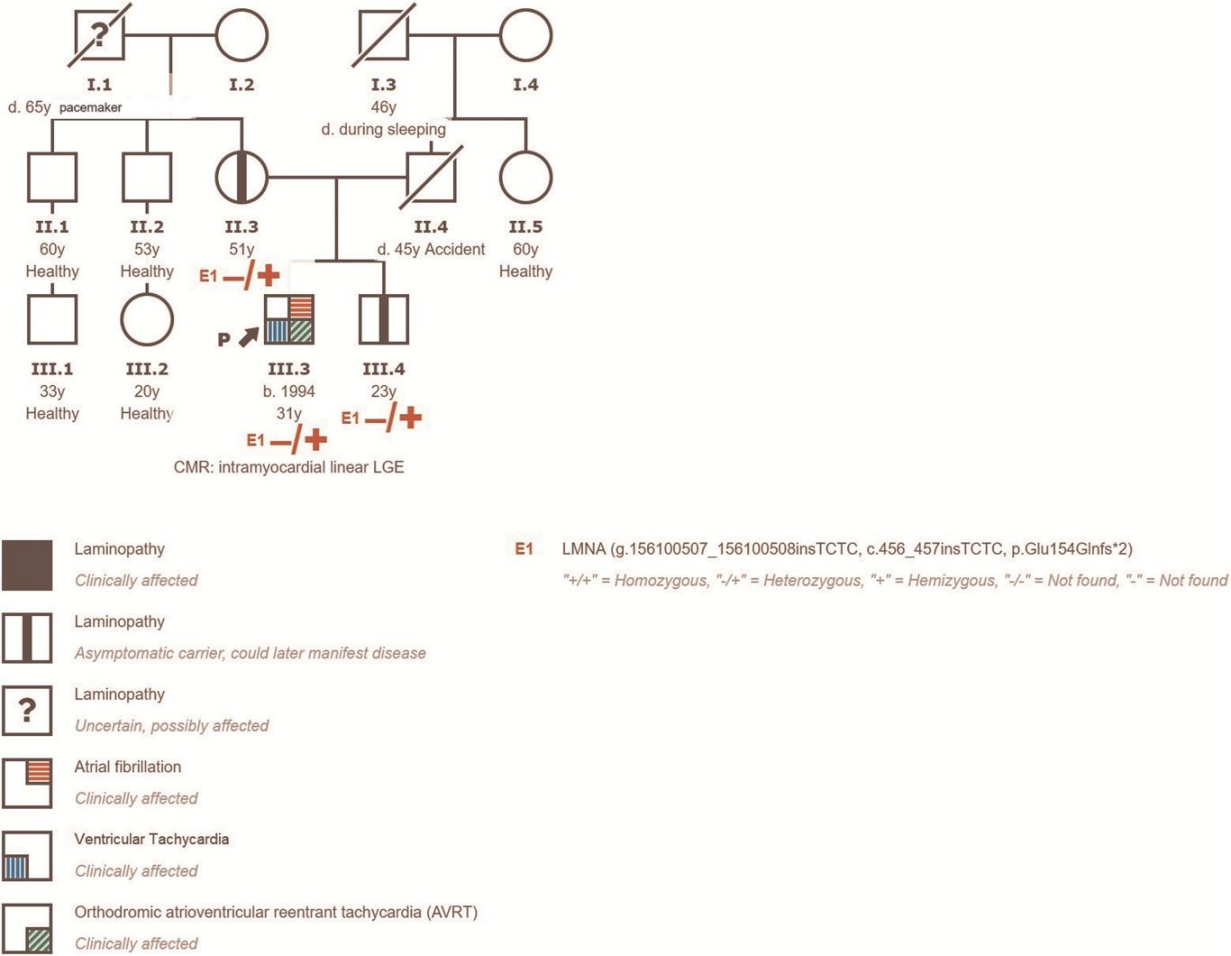
The pedigree of a family with an LMNA gene mutation.

One year after RFA of slow pathway CMR revealed a slight increase in LV (145 mL) and RV (129 mL) end-diastolic volumes, along with a continued trend of declining EF in both ventricles (LVEF: 57%, RVEF: 40%), and a new intramyocardial linear LGE was observed along the anteroseptal segment, extending into the anterior segment (*Figure 2B*) and (see *Supplements S2* and *S3*). Detailed pedigree evaluation (*Figure 3*) revealed that the patient’s grandfather had pacemaker implanted at middle age and died due to ‘cardiac problems’ at age 65.

The patient was referred for genetic consultation,^8^ and comprehensive genetic testing showed that the patient is a heterozygous carrier of the NM_170707.3.456_457insTCTC, NP_733821.1.Glu154GlnfsX2 variant in the LMNA gene. This variant has been classified as likely pathogenic and is linked to laminopathy development based on current evidence, ACMG classification standards, and ClinGen adaptations. The criteria used were PM2_supporting and PVS1. This variant has not been reported before. It is absent from the gnomAD v4.1.0 database and has not been found in the proprietary database of the international genetic laboratory, Health in Code (Spain), which includes over 45 000 patients. This four-nucleotide insertion in the LMNA gene causes a frameshift (out-of-frame), resulting in a null variant. Variants such as nonsense, frameshift, splice-site, and structural variants in the LMNA gene usually lead to loss of protein function, which is a well-established pathogenic mechanism linked to disease development.

Genetic family screening identified the same variant in the asymptomatic patient’s mother and the patient’s brother.

According to the online calculator for the 5-year risk of VT in LMNA mutation carriers, our patient has 10.8% risk,^9^ and the implantation of a cardioverter–defibrillator for primary prophylaxis of SCD should be considered.^9^

## Discussion

Atrioventricular re-entrant tachycardia and AVNRT are generally considered rather sporadic. The molecular and genetic factors underlying re-entrant SVT remain largely unknown. There are a few reports of familial Wolff–Parkinson–White (WPW) syndrome, with the *PRKAG2* gene being the most well-characterized contributor.^10^ Mutations in SCN1A and RYR2 have been linked to AVNRT, although no cases involving *LMNA* gene mutations have been described thus far.^11^

In our patient, the coexistence of AVRT and, likely, AVNRT with other arrhythmias (inducible subclinical AF and non-sustained VT) raised suspicion of structural heart disease, which led to echocardiographic review and more detailed imaging with CMR. Presumed myocarditis may have reflected the inflammatory phase of cardiomyopathy, including a laminopathy, or been caused by a virus, as well as other conditions.^5,8,12^ Within 3 years of follow-up, subsequent CMR and genetic evaluation revealed evidence of a genetic cardiomyopathy (GCMP).

### Our case raises important questions

Might early genetic screening in reciprocating tachyarrhythmia patients facilitate timely diagnosis and appropriate management of GCMP, potentially improving outcomes in individuals at risk for HF and SCD? Or, in other words, what if coexistent AVRT and dual AV nodal physiology could be early indicators of the specific LMNA-related cardiomyopathy?

Genetic testing in revealing early cardiomyopathic changes warrants consideration. Recent genome-wide association studies in AVNRT patients have identified genetic variants near genes previously linked to increased susceptibility to HF and relatively common dilated cardiomyopathy.^13^ These findings suggest that AVNRT may fall within the broader spectrum of atrial cardiomyopathy and, in some cases, represent an early manifestation of cardiomyopathy or HF.^14^

While RT patients typically exhibit minimal structural or inflammatory abnormalities, the potential role of further CMR deterioration played a crucial role in clinical comprehension of the case. A fibrosis, as well as an inflammation may indeed be present in some SVT patients, and both may contribute to the re-entrant substrate.

So, should genetic screening be broadly implemented in patients with SVT without overt structural heart disease? The answer is likely no for the general population. However, in SVT patients who also exhibit multiple arrhythmias, a history of RT and their recurrence following RFA, suspicious family history, and imaging with subtle evidence of dynamic structural abnormalities, the answer is more likely yes.

## Conclusion

This case describes a common RT occurring in the context of a rare GCMP. While the association appears coincidental and lacks established aetiological links, it underscores the need for vigilance in identifying potentially life-threatening genetic disorders in patients with SVT. The presence of a concerning family history or abnormal visual findings should prompt comprehensive CMR imaging and genetic evaluation.

## Lead author biography


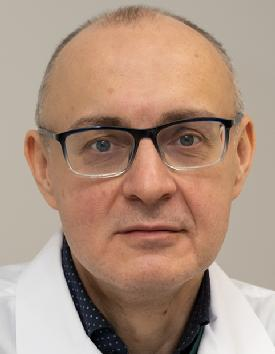


Dr Evgeny Zhelyakov is cardiologist and electrophysiologist at Pirogov Russian Research Medical University in Moscow, Russia.

## Supplementary Material

ytaf417_Supplementary_Data

## Data Availability

The data underlying this article are available in the article and in its online Supplementary material.
