# Supplementary material for: Reciprocated tachycardias in cardiac laminopathy: a clinical case report
Source: Eur Heart J Case Rep. 2025 Aug 28;9(9):ytaf417. doi: 10.1093/ehjcr/ytaf417 (PMC12415688; doi:10.1093/ehjcr/ytaf417)
Supplement: ytaf417_Supplementary_Data [file ytaf417_supplementary_data.zip › legenda Supplement (1).docx]

**Supplement 1.** Baseline 12 leads ECG

**Supplement 2.** CMR images with conventional and parametric mapping techniques in 2022 (A) and 2024 (B). T2 mapping images remained without myocardial edema (left site, panels A and B). Native T1 mapping with normal T1 relaxation time within two years (middle, panels A and B). Phase sensitive inversion recovery CMR image demonstrating LGE involving the sub-epicardium in the posterolateral wall (white arrows) in 2022 (right site, panel A) and a new intramyocardial linear LGE along the anteroseptal segment, extending into the anterior segment (grey arrows) in 2024 (right site, panel B).

**Supplement 3.** CMR conventional Steady-state free precession (SSFP) cine imaging in the four-chamber plane in 2022 (A) and 2024 (B). Within two years CMR revealed slight increase in LV (from 122 to 145 ml) and RV (from 125 to 129 ml) end-diastolic volumes, along with a continued trend of declining EF in both ventricles (LVEF: from 65 to 57%, RVEF: from 47 to 40%)
