# Supplementary figures and images for: Reciprocated tachycardias in cardiac laminopathy: a clinical case report
Source: Eur Heart J Case Rep. 2025 Aug 28;9(9):ytaf417. doi: 10.1093/ehjcr/ytaf417 (PMC12415688; doi:10.1093/ehjcr/ytaf417)

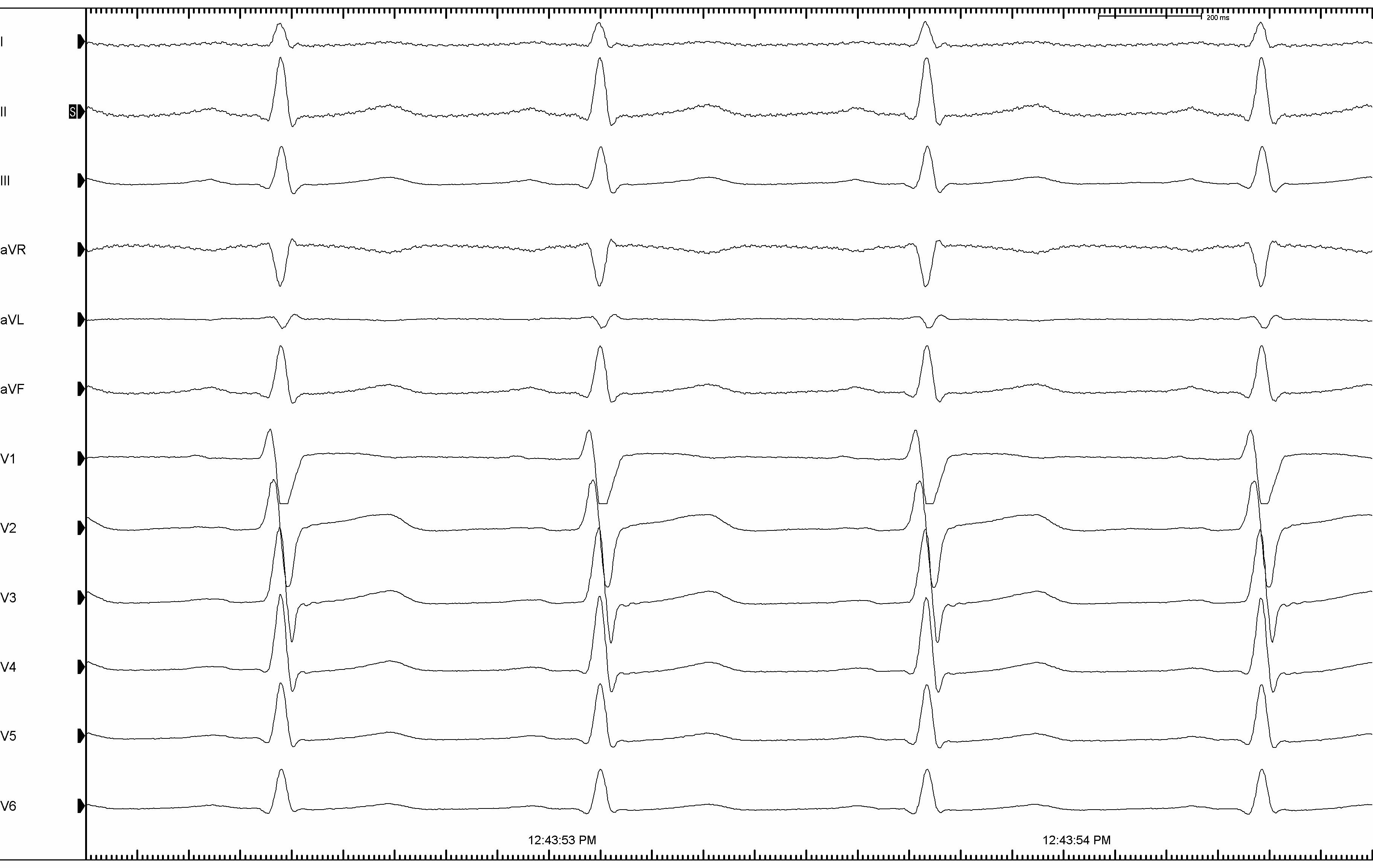

Supplement: ytaf417_Supplementary_Data [file ytaf417_supplementary_data.zip › Supl12leadECG.jpg]

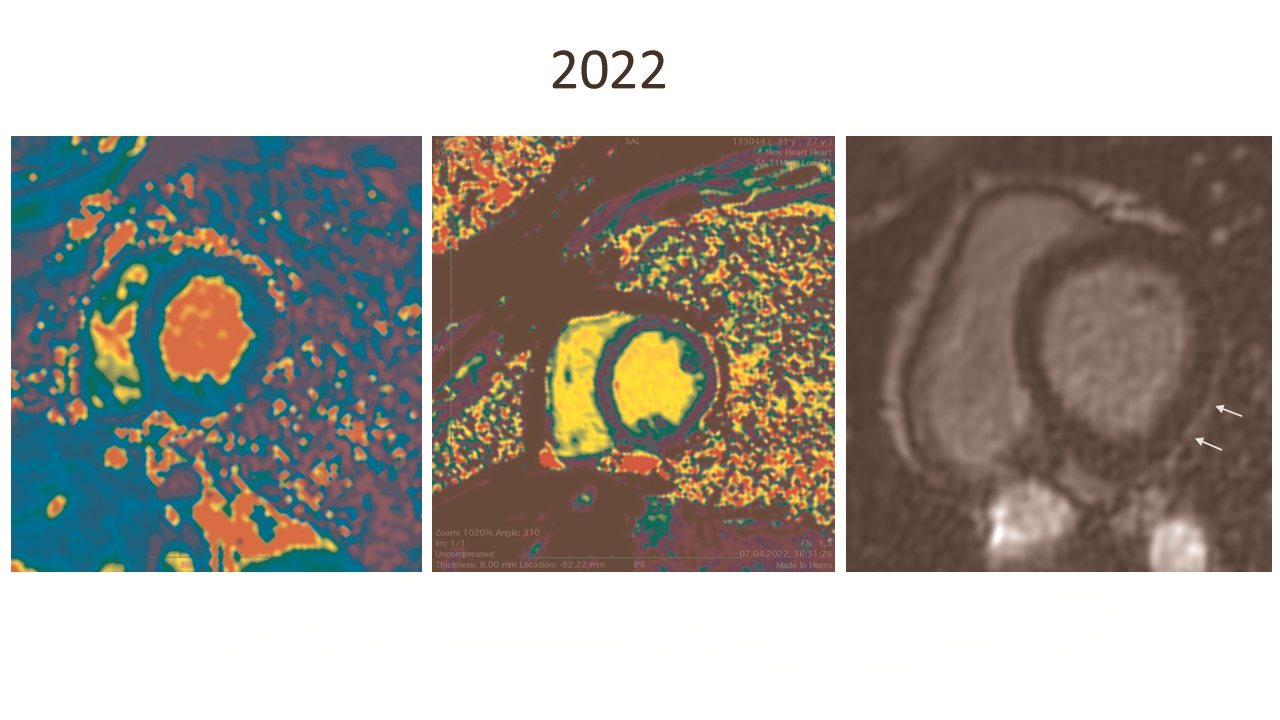

Supplement: ytaf417_Supplementary_Data [file ytaf417_supplementary_data.zip › supl2A300dpi.tif]

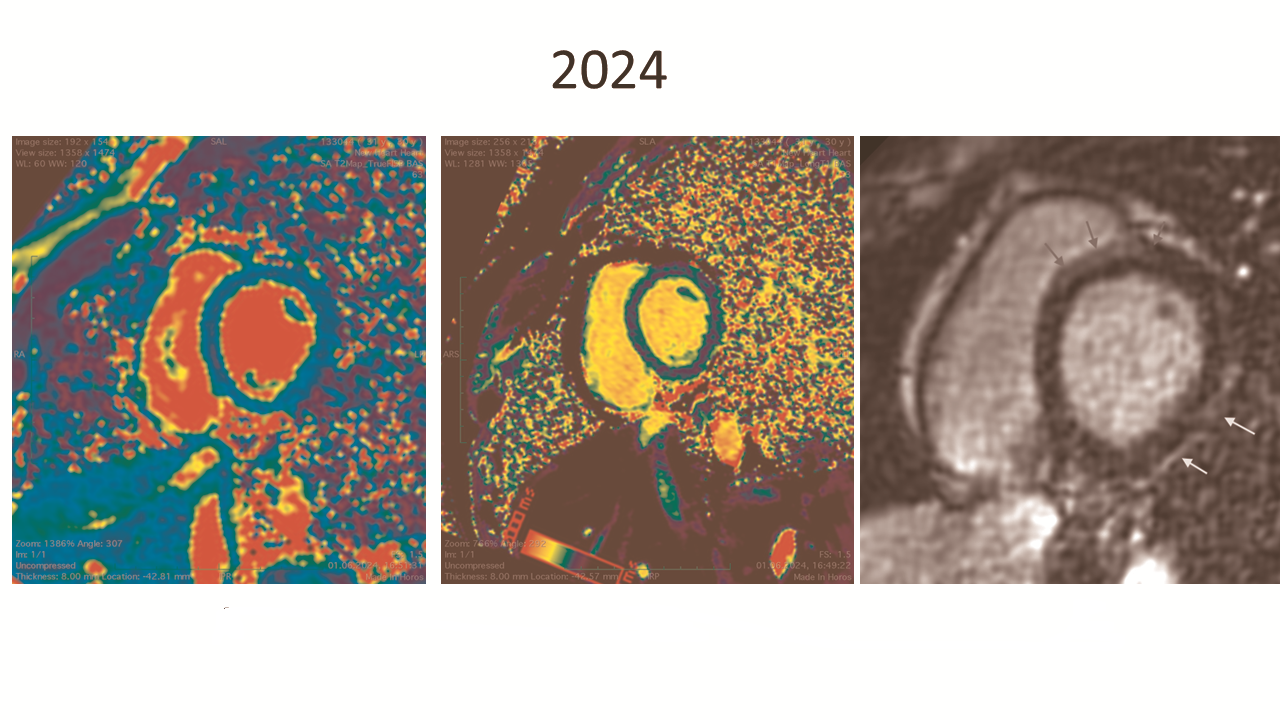

Supplement: ytaf417_Supplementary_Data [file ytaf417_supplementary_data.zip › supl2B300dpi.tif]
